# Paris Green Poisoning Case

**Published:** 1877-11

**Authors:** E. J. Bowman

**Affiliations:** Neffsville, Pa.


### PARIS GREEN POISONING.

From the *Medical Brief.*

Upon the morning of July 3d, 1877, at 10 o'clock A. M., I was called to attend a child, one and a half years of age, that had taken about a tablespoonful of pure Paris-green. With the messenger I sent an emetic; and when I arrived the child was vomiting freely. After the vomiting ceased, I administered an active purgative; when the bowels moved, the fœces were mixed with Paris-green. Not having the regular antidote prepared, I gave the child a teaspoonful of the ferri sub-carbonas, teaspoonful doses, every hour. I now left the patient. I saw it again in the evening. No bad symptoms. Continued the iron, as before. Saw the child again on the morning of the 4th. passed urine; stained linen very green, showing absorption. Finding no bad symhtoms, I continued the iron, and the child recovered perfectly on the fifth day. E. J. BOWMAN.

Neffsville, Pa., July 23d, 1877.

